# Structural genomics analysis of uncharacterized protein families overrepresented in human gut bacteria identifies a novel glycoside hydrolase

**DOI:** 10.1186/1471-2105-15-112

**Published:** 2014-04-17

**Authors:** Anna Sheydina, Ruth Y Eberhardt, Daniel J Rigden, Yuanyuan Chang, Zhanwen Li, Christian C Zmasek, Herbert L Axelrod, Adam Godzik

**Affiliations:** 1 Joint Center for Structural Genomics, 10550 North Torrey Pines Road, BCC-206, La Jolla, California 92037, USA; 2 Bioinformatics and Systems Biology Program, Sanford-Burnham Medical Research Institute, La Jolla, CA 92037, USA; 3 Wellcome Trust Sanger Institute, Wellcome Trust Genome Campus, Hinxton, Cambridgeshire CB10 1SA, UK; 4 European Molecular Biology Laboratory, European Bioinformatics Institute, Wellcome Trust Genome Campus, Hinxton, Cambridgeshire CB10 1SD, UK; 5 Institute of Integrative Biology, University of Liverpool, Crown Street, Liverpool L69 7ZB, UK; 6 Stanford Synchrotron Radiation Lightsource, Menlo Park, CA 94025, USA; 7 Center for Research in Biological Systems, University of California, 9500 Gilman Dr., La Jolla, CA 92093-0446, USA

**Keywords:** Glycoside hydrolase, Carbohydrate metabolism, 3D structure, Protein family, Protein function prediction, Domain of unknown function, DUF

## Abstract

**Background:**

*Bacteroides spp.* form a significant part of our gut microbiome and are well known for optimized metabolism of diverse polysaccharides. Initial analysis of the archetypal *Bacteroides thetaiotaomicron* genome identified 172 glycosyl hydrolases and a large number of uncharacterized proteins associated with polysaccharide metabolism.

**Results:**

BT_1012 from *Bacteroides thetaiotaomicron* VPI-5482 is a protein of unknown function and a member of a large protein family consisting entirely of uncharacterized proteins. Initial sequence analysis predicted that this protein has two domains, one on the N- and one on the C-terminal. A PSI-BLAST search found over 150 full length and over 90 half size homologs consisting only of the N-terminal domain. The experimentally determined three-dimensional structure of the BT_1012 protein confirms its two-domain architecture and structural analysis of both domains suggests their specific functions. The N-terminal domain is a putative catalytic domain with significant similarity to known glycoside hydrolases, the C-terminal domain has a beta-sandwich fold typically found in C-terminal domains of other glycosyl hydrolases, however these domains are typically involved in substrate binding. We describe the structure of the BT_1012 protein and discuss its sequence-structure relationship and their possible functional implications.

**Conclusions:**

Structural and sequence analyses of the BT_1012 protein identifies it as a glycosyl hydrolase, expanding an already impressive catalog of enzymes involved in polysaccharide metabolism in *Bacteroides* spp. Based on this we have renamed the Pfam families representing the two domains found in the BT_1012 protein, PF13204 and PF12904, as putative glycoside hydrolase and glycoside hydrolase-associated C-terminal domain respectively.

## Background

The human gut microorganisms form a specialized community, the human gut microbiome, that plays an important role in normal functioning of digestive metabolism, in nutrition and, possibly, in the development of the human immune system [1]. As part of their adaptation to the gut environment, the bacterial species forming the microbiome have developed an extensive ability to metabolize a wide variety of polysaccharides. This allows humans to utilize a broad range of plant- and host-secreted glycans that would otherwise be indigestible. *Bacteroides spp.* are an essential part of the human gut microbiome and provide us with a broad range of metabolic enzymes [2,3]. The Gram-negative bacterium *Bacteroides thetaiotaomicron* is a dominant member of the normal human distal intestine and colon microbiota and has a large repertoire of genes for harvesting nutrients from a wide range of polysaccharides derived from both plants as well as hosts [4].

BT_1012 from *Bacteroides thetaiotaomicron* VPI-5482 is a protein of unknown function and a member of a large family of uncharacterized proteins. The sequence analysis predicted that the BT_1012 protein is related to glycoside hydrolase family 5, based on the Carbohydrate-Active Enzymes (CAZy) classification [5]. A classification of glycoside hydrolases into families based on amino acid sequence similarity has been in place for a few decades [6]. However, structure analysis and comparisons allow us to confirm and fine tune function predictions based on sequence analysis.

A search against Pfam database [7] predicts that this protein has two domains: the N-terminal domain belongs to the PF13204 (DUF4038) family and the C-terminal domain, which is a member of the PF12904 family, currently annotated as a collagen-binding domain. Many protein families annotated as DUFs represent divergent branches of already known and well-characterized families, and the DUF4038 is no exception. It belongs to the Pfam clan CL0058, the TIM barrel glycosyl hydrolase superfamily. This allows us to hypothesize that it also may be a carbohydrate hydrolase.

The Pfam database currently contains over 3,500 families annotated only as “domains of unknown function” [8]. Such families, because of the acronym of their name as known as DUFs and are differentiated by their number, such as DUF4038. In a coordinated effort the four large-scale centers of the NIH Protein Structure Initiative have determined the first three-dimensional structures for representatives of more than 400 of such families, and the first 250 were analyzed by our group previously [9]. In this paper we analyze the crystal structure of the BT_1012 protein and combine several bioinformatics approaches to suggest the function of this protein. The structure the BT_1012 protein was solved by JCSG and deposited in the PDB database as [PDB: 3KZS] in 2009.

## Results and discussion

### Structural determination

The crystal structure of the BT_1012 (NP_8009925.1) protein from *Bacteroides thetaiotaomicron* VPI-5482 was determined to 2.1 Å by MAD (Multi-wavelength anomalous diffraction) phasing. Data-collection, model, phasing, and refinement statistics are summarized in Additional file 1: Table S1. The final model includes four molecules (residues 27–483), sixteen sulfate ions, two (4S)-2-methyl-2,4-pentanediol (MPD), eight (4R)-2-methylpentane-2,4-diol (MRD), and 1208 water molecules in the asymmetric unit. Modeling of the electron density for 2-methylpentanve-2,4-diol was subjective because of the 2.10 Å resolution limit, and further analysis showed that either the R or S enantiomer of 2-methylpentane-2,4-diol could be modeled and refined. The structure is composed of twelve alpha-helices, five 310-helices, twenty beta strands. Gly 0 (which remained at the cleavage of the expression/purification tag), the region from Ala 22-Thr 26 on subunits A, C, and D; and Ala 22-Gln 27 on subunit B were disordered and not modeled. Subunit D was partly disordered in the asymmetric unit and its statistics are slightly different from that of subunits A-C. The Matthews coefficient (VM: Matthews, 1968) is 2.77 Å3Da-1 and the estimated solvent content is 55.6%. The Ramachandran plot produced by MolProbity [10] shows that 94.1% with seven outliers.

**Figure 1:**
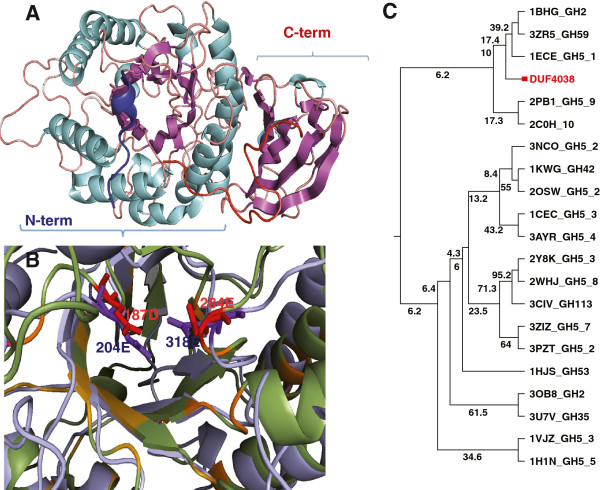
**A ribbon diagram of the BT_1012 (3KZS) structure, active site and homologs of DUF4038**. Panel **A**. The ribbon diagram of the BT_1012 (3KZS) structure, with alpha helixes shown in light blue, beta-sheet in purple. N-terminal is identified as a dark blue helix, C-terminal end as a red coil. N-terminal domain covers residues 27–370, C-terminal domain, 371–483. Panel **B**. Superimposition of the N-terminal domain of the BT_1012 (3KZS) structure, corresponding to the DUF4038 domain (green) and the family GH5 endo-beta-mannanase from *Lycopersicon esculentum* (tomato) (PDB 1RH9) (blue), active sites residues colored as red and purple, respectively. Panel **C**. Phylogenetic tree based on FFAS alignment of DUF4038 homologs from different glycoside families.

The crystal structure of BT_1012 consists of two domains: the N-terminal (β/α)8−barrel (ΤΙΜ-barrel) domain comprising residues 27 to 370 and the C-terminal Greek-key β-sandwich domain covering residues 371 to 483, respectively. FastSCOP classification database search results confirms the TIM beta/alpha barrel fold classification of N-terminal domain shows and further identifies it as a member of the beta-glycanases SCOP superfamily, supporting the notion that it has a function in carbohydrate metabolism. The structure of the C-terminal domain is similar to the C-terminal domain of a human alpha-galactosidase (PDB code 1R46), indicating that it might be involved in carbohydrate binding (Figure 1). Families describing both domains of this protein are strongly overrepresented in human gut metagenomic datasets. For instance, PF13204 family has 345 and PF13204 has 120 homologs in the UniProt UniProtKB database [11], while the METAHIT (Metagenomics of the Human Intestinal Tract) dataset [12] has 1155 and 514 homologs respectively, despite being four times smaller than UniProt UniProtKB (Chang Y, Jaroszewski L, Godzik A: Analysis of expanded repertoire of protein families in human gut microbiome, in preparation.) It is very unlikely for BT_1012 to be involved in collagen binding, as suggested by some database annotations. Detailed analysis for each domain follows.

### N-terminal domain

**Figure 2:**
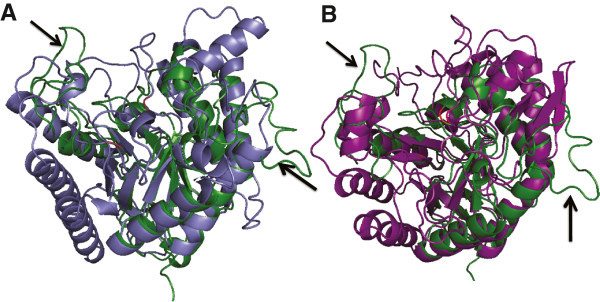
**A comparison of loops in the BT_1012 (3KZS) structure and mannanase enzymes.** Structure alignment of the N-terminal domain of the BT_1012 (3KZS) structure, corresponding to the DUF4038 domain (green) and the family GH5 endo-beta-mannanase from *Lycopersicon esculentum* (tomato) (PDB 1RH9) (blue), (Panel **A**) and *Chrysonilia sitophila* endo-beta-D-1,4- mannanase (4AWE) colored purple (Panel **B**). Black arrows shows extended loops. The active site is shown in red. According to the database of secondary structure assignments (DSSP) the N-terminal domain of the BT_1012 (3KZS) has 51% of coil secondary structure, compared to 39% in 1RH9 45% in 4AWE.

The N–terminal domain of of the BT_1012 protein is the first experimentally determined structure of a representative of Pfam family PF13204. This family belongs to a large superfamily containing a range of glycoside hydrolase enzymes with a TIM barrel fold (Pfam clan CL0058). This Pfam clan includes members of the following CAZy clans: GH-A, GH-D, GH-H and GH-K. A sequence similarity search by PSI-BLAST and FFAS against PDB found that the most similar proteins were endo-beta-1,4-mannanases from the GH5 family: 3ZIZ, 4AWE and 1QNO (sequence identity 15%, Additional file 2: Table S2). The most similar structures identified by DALI and FATCAT (both in rigid and flexible alignment modes) are also endo-beta-mannanase enzymes, but from the GH5 family (Additional file 2: Table S2). Although the PF13204 family is predominantly prokaryotic, most of the structures identified in the DALI and FATCAT search belong to eukaryotes, mostly fungi and plants, reflecting lack of structural studies of proteins from this clan in bacteria. Structural alignment of the PF13204 domain with endo-beta-mannanase (PDB ID: 1RH9, Z–score 19, 17% sequence identity) reveals that the putative active site has Asp187 as the catalytic nucleophile/base residue of the active site and Glu284 as the catalytic proton donor position instead of the highly conserved Glu - Glu pair found in the GH-A superfamily and in particular in GH5 (Figure 1B). However, the loops that form the presumed active site are quite a bit longer than those found in many mannanase enzymes (Figure 2), suggesting significant differences in binding specificity. In the cup-shaped region of the putative active site, we found highly conserved Trp63, Trp149 and Tyr287 residues which are very likely involved in carbohydrate binding (Figure 1B). The role of aromatic amino acids in carbohydrate binding was established decades ago [3]. The aromatic residues (tryptophan, tyrosine and, less commonly, phenylalanine) form the hydrophobic platforms in the binding sites, which adopt different shapes in order to interact with a variety of carbohydrates [13].

**Figure 3:**
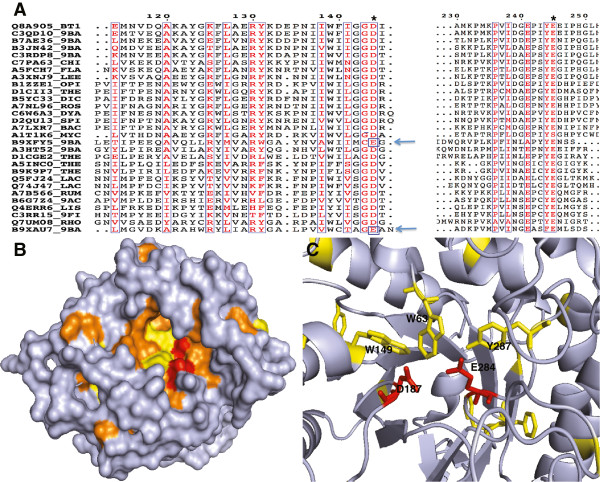
**Residue conservation in the PF13204 family (DUF4038).** (Panel **A**) The sequence conservation of the PF13204 seed alignments by ESPirit. The black arrows show the substitution in the active site, which marked by asterisks (Panel **B**). The surface of the N-terminal domain of the BT_1012 (3KZS), corresponding to the DUF4038 domain, conserved residues colored orange or yellow for aromatics, the putative active site colored red (Panel **C**). The ribbon diagram of the putative active site, the closest aromatic residues colored yellow and shown as sticks.

Sequence conservation analysis was performed to identify residues that may be functionally important. We found that the potential active site of this domain is more divergent than in any of the subfamilies of the GH5 family. The GH5 family has about 98% conservation of catalytic residues of the active site (Glu) while proteins in the PF13204 family has Asp at the catalytic site in 82% of cases, Glu in 17.3% and Asn in the remaining 0.7%. To understand the possible functional role of other conserved residues, we show them on the surface of the protein model (Figure 3). Most of the conserved residues formed a cleft on one side of the protein near the active site, while the opposite side has just a few conserved exposed residues. The cleft has conserved aromatic residues: Tyr166 and Tyr174 (Figure 3, colored yellow). This supports the hypothesis that this domain may have a carbohydrate binding function.

**Figure 4:**
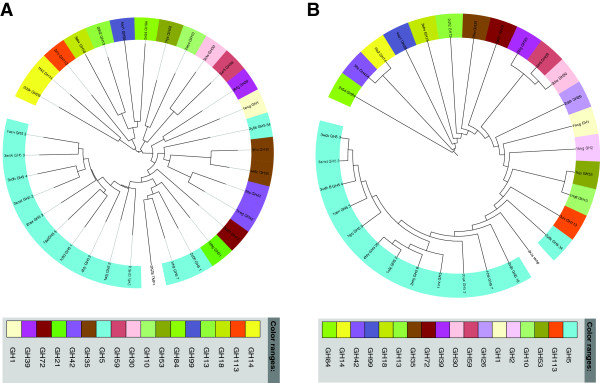
**Structure similarity trees of TIM barrel glycosyl hydrolases.** The tree based on DALI (Panel **A**) and POSA (rigid alignment model) (Panel **B**) alignments of the N-terminal domain of the BT_1012 (3KZS) structure, corresponding to the DUF4038 domain.

A phylogenetic tree based on all members of the PF13204 family shows that the members that have Glu in the catalytic site do not form a single branch in the tree (Additional file 3: Figure S1). Because of a low sequence similarity to other glycoside hydrolases, we built trees based on DALI structural alignments [14] and using POSA (Partial Order Structure Alignment) [15] (Figure 4). Briefly, POSA calculates a distance matrix using the P-value of the similarity of two structures from FATCAT structural alignment algorithm with the rigid alignment option and then performs a single linkage clustering on the distance matrix to generate the tree [15]. Both trees support classification of PF13204 family with the GH5 family. We also performed a phylogenetic analysis based on the alignments obtained by FFAS (a profile–profile alignment algorithm, which uses a fold recognition tool and is more sensitive than the popular sequence–profile matching PSI-Blast algorithm) [16]. This analysis supports the hypothesis that PF13204 and the GH5 family might have the same ancestor (Figure 1C). However, the GH5 family has a strongly conserved active site, which is not the case for the PF13204 family. Taken together, we therefore surmise that the PF13204 family is not a part of the GH5 family but is a first representative of a novel, albeit sharing a common ancestor with GH5 family, glycoside hydrolase family, and we have renamed the Pfam domain as a putative glycoside hydrolase. This new family would expand an already impressive catalog of 394 carbohydrate-hydrolytic enzymes, which mostly represent GT2 (Glycosyl transferase family 2, 39 members), GH43 (Glycoside hydrolase family 43, 34 members) and GH2 (Glycoside hydrolase family 2, 32 members) families.

### C-terminal domain

The C–terminal domain of the BT_1012 protein, corresponding to the Pfam PF12904 domain family, was previously annotated in Pfam as a collagen-binding domain. This domain has a beta-sandwich fold (Figure 1A). According to Pfam, this domain is found almost exclusively at the C–termini of proteins with the PF13204 domain. The structural comparison by DALI and FATCAT found that this domain to be similar to C-terminal domains of different hydrolases with a broad spectrum of substrate specificity (for example galactosidase, xylosidase, and dextranase). Based on CAZY classification, the top hits of FATCAT and DALI searches are proteins bearing catalytic domains belonging to GH27 (GH-D), GH39 (GH-A), and GH59 (GH-A) (Additional file 2: Table S2) glycosyl hydrolase families. The top ten DALI and FATCAT hits are different from those found in the N-terminal domain search (Additional file 2: Table S2) and these proteins do not have similar domain combinations. It is interesting that several proteins with overall structural similarity to 3KZS (over the entire length of the structure) belong to cellulases of subfamilies 8 and 34 of the GH5 family (Additional file 4: Table S4). The GH5 family has different carbohydrate-binding modules (CBM), however some of them (CBM6, CBM15, CBM29) have beta-sandwich folds [17]. According to the CAZy database, 16 of the 394 *B. thetaiotaomicron VPI-5482* enzymes possess CBM domains. Of these, the majority (11) belong to the CBM32 family, which also has a beta-sandwich fold.

**Figure 5:**
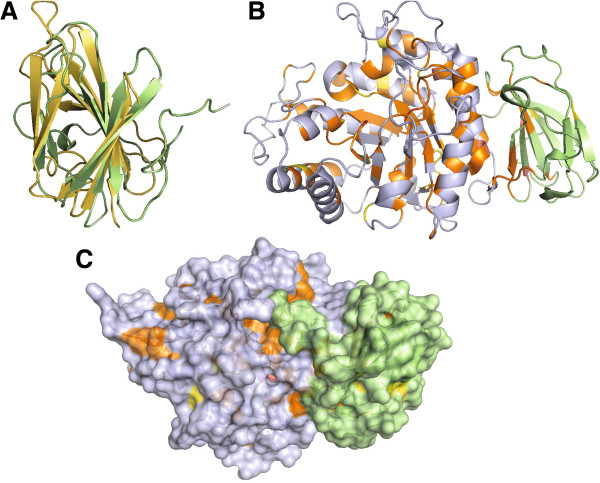
**A residue conservation analysis for the BT_1012 (3KZS) structure.** (Panel **A**) A superposition of the C-terminal domain (limegreen) with human alpha-galactosidase (1R46) colored dark yellow. (Panel **B**) A ribbon diagram and (Panel **C**) the surface of the BT_1012 (3KZS) structure shows conserved residues colored orange and yellow (aromatic); DUF4038 colored in blue, C-terminus in green.

A putative role for the C-terminal domain as a CBM would be strengthened by any observation of conserved, solvent-exposed aromatic residues [13] but none were apparent. Nevertheless, there is a conserved surface patch composed of Asn385, Aln394, Arg396, and Asn443, which could have a substrate binding function (Figure 5). No conserved aromatic residues are present on the surface. Moreover, the C-terminal domain of the BT_1012 protein is on the opposite side of the putative active site and interacts strongly with the TIM barrel domain, which provides Tyr312, Phe316, Phe369 and Pro370 to this interaction. At the same time, most of the domains identified in the structural similarity search with the C-terminal domain of the BT_1012 protein do not bind carbohydrates, despite being part of glycolytic enzymes. Instead they have auxiliary function; for example stabilization of catalytic domains or involvement in domain-domain interaction (Additional file 5: Table S3). To sum up, this domain more is likely to stabilize the N-terminal domain than be directly involved in substrate binding.

### The whole structure 3KZS and functional prediction for each domain

The structure similarity searches using DALI and FATCAT with the whole BT_1012 structure still identified single domain proteins as the top hits. Thus we separately analyzed top multi-domain proteins found in these searched (Additional file 4: Table S4). With this constraint, the top match is the beta-galactosidase from *Bacillus circulans* sp. alkalophilus (PDB: 3TTS). This enzyme has three domains and an atypical active site [18]. According to the authors, the function of the third domain, which has a beta-sandwich fold, is purely structural because they did not find clefts on the surface or cavities that could have carbohydrate binding function.

The distant homology recognition for the BT_1012 protein by FFAS found that the top ten hits belong to the GH5 family. All other GH5 hits have a single (catalytic) domain with only one exception - arabinoxylan-specific xylanase from *Clostridium thermocellum* ATCC 27405 (PDB: 2Y8K) has an additional domain that belongs to the CBM6 family. This module was shown to increase thermostability of the catalytic domain and was involved in binding of cellohexaose or xylohexaose [19]. Another example of a glycoside hydrolase with an additional C-terminal domain that is a distant homolog of 3KZS is β-xylosidase II from *Caulobacter crescentus* CB15 (PDB: 4EKJ). This protein belongs to the GH39 family and has a C terminal domain that regulates the accessibility and molecular topography of the active site [20]. Thus, there are several examples that support our hypothesis that the C-terminal domain has a supportive and regulatory function. This domain is always found in two domain proteins following the PF13204 domain, and about 60% of the PF13204 family members have the C-terminal domain (see more examples in Additional file 4: Table S4).

Many carbohydrate-related proteins in Bacteriodes are grouped into polysaccharide utilization loci, often of well-defined induction specificity. However, the BT_1012 coding gene is not a part of any of these loci. Genomic-context analysis using the MicrobesOnline database [21] and STRING revealed that BT_1012 is colocalized with alpha-rhamnosidase, and rhamnosidases (NOG10735 on STRING) are linked by genome neighborhoods in other species too. Rhamnose is commonly bound to other sugars, but is also a common component of plant glycosides [22]. This suggests that BT_1012 may be a partner of alpha-rhamnosidase in plant sugar degradation. Taken together that the catalytic domain has longer loops compared to mannanases (for example 1RH9 and 1UUQ), this may suggest that it can make additional interactions with long polysaccharide substrates.

**Figure 6:**
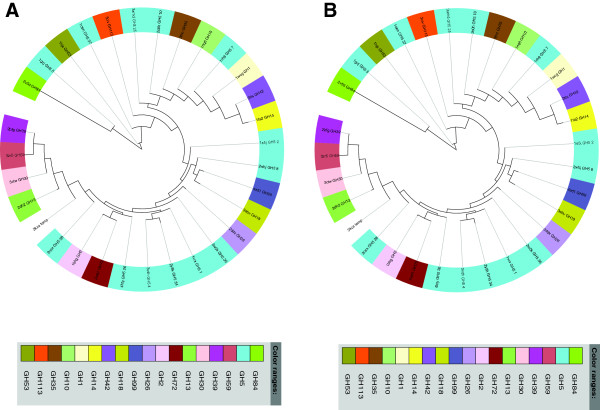
**Structure similarity tree of 3KZS.** A tree based on DALI (Panel **A**) and POSA (rigid alignment model) (Panel **B**) alignments of 3kzs coordinates.

Phylogenetic analysis did not find any correlation with the presence of the C-terminal domain and having Glu/Asp in the catalytic site. However, we found that Glu/Asp members form three different branches (Additional file 3: Figure S1). The C-terminal domain may change the conformation of the central catalytic domain and provide the enzyme with a broad spectrum of substrate specificity. The structure similarity tree built by POSA (Figure 6) shows that 3KZS has groups closely with GH5 family. In dbCAN [23], the BT_1012 was identified as a subfamily 38 of the GH5 family. However, this database was developed to provide a capability for automated CAZyme signature domain-based annotation for any given protein data set [23], thus we can question this annotation. Moreover, the tree built based on DALI alignment shows that 3KZS is close to GH13 and GH14 families and distant from GH5 family (Figure 6). Taking into account that GH5 is mostly represented by enzymes having a single catalytic domain, with strong conservation of Glu at the active sites and distant sequence similarity of PF13204 and particular 3KZS, we conclude that BT_1012 is the a novel two-domain glycoside hydrolase with a catalytic domain and a C-terminal auxiliary domain.

## Conclusions

The crystal structure of the BT_1012 protein and structure-based sequence-structure-function analysis suggests that BT_1012 and its approximately 150 full length homologs, ranging in sequence identity from 40-60%, are two domain glycoside hydrolases, which include an N-terminal catalytic domain and a C-terminal auxiliary domain which may be involved in stabilizing or regulating the catalytic domain. Based on our findings, we have renamed the PF13204 family as “putative glycoside hydrolase” and the PF12904 family as “glycoside hydrolase-associated C-terminal domain”.

## Methods

Protein production and crystallization of CA_C2195 was carried out by standard JCSG protocols [24]. Data collection was performed at SSRL beamline 11–1. The crystal structure was determined by multi-wavelength anomalous diffraction phasing (MAD) using seleno-methionine-derivatized protein and x-ray data collection, processing, structure solution, tracing, crystallographic refinement, and model building were performed using BLU-ICE [25], MOSFLM [26]/SCALA [27], SHELXD [28]/AUTOSHARP [29], ARP/wARP [30], REFMAC [31], and COOT [32]. Modeling, phasing, and refinement statistics were done based on the standard JCSG protocol [33-36]. After building and refining the protein chains A, B, and C, anomalous difference Fourier maps and isomorphous difference Fourier maps suggested that there was a fourth subunit in the crystallographic asymmetric unit. However, the electron density for this subunit is poor, and both the electron density map and anomalous difference Fourier maps indicate that this extra subunit is disordered. The anomalous difference Fourier peaks were used as a guide the building of chain D. The pattern of these peaks supports modeling of the subunit in two half occupancy conformations. Note that while chain D part B would symmetry clash with itself, it does not clash with the symmetry mate of part A. Additionally, chain D part A does not clash with the symmetry mate of chain D part B.

To find homologs for sequence conservation analysis, PSI-BLAST was used to search the Uniref90 database in 3 iterations with e-value cutoff of 0.0001, identifying 150 homologs with sequence similarity between 35-95%. MAFFT was used for multiple alignment [37]. Figures were prepared using PyMOL [38] and ESPirits [39]. The protein secondary-structure elements were determined according to the database of secondary structure assignments (DSSP) [40]. Phylogenetic analysis was performed using distance based approaches, such as FastM 1.1 [41], neighbor-joining from PHYLIP 3.66 [42] pair-wise distances were calculated by TREE-PUZZLE 5.2 using the VT model [43]. A phylogenetic tree was drawn and visualized with FORESTER [44]. Pfam data is from release 27.0 [7].

## Competing interests

The authors declare that they have no competing interests.

## Authors’ contributions

AS conceived the study, analyzed the results and wrote the manuscript. RYE contributed her expertise in the data analyses and revisions of the paper. DJR contributed his expertise in structural analysis. YC did the structure description. ZL built the POSA tree. CZ contributed his expertise in phylogenetic analysis and proofreading. HLA refined the 3KZS structure and helped with the structure description. AG conceived the overall idea, coordinated the collaboration, guided the progress of this study and contributed to the writing of the manuscript. All authors read and approved the final manuscript.

## Supplementary Material

Additional file 1: Table S1 Data collection and refinement statistics (PDB 3kzs). Values in parentheses are for the highest resolution shell. ^†^*R*_*merge*_ = Σ_*hkl*_Σ_*i*_|*I*_*i*_*(hkl) - (I(hkl))*|/Σ_*hkl*_ Σ_*i*_*(hkl)*. ^‡^*R*_*meas*_ = Σ_*hkl*_[*N/(N*-1)]^1/2^Σ_*i*_|*I*_*i*_*(hkl) - (I(hkl))|/*Σ_*hkl*_Σ_*i*_*I*_*i*_*(hkl)*[33]. ^‡‡^*R*_*p.i.m*_ (precision-indicating *R*_*merge*_) = Σ_*hkl*_[(1/(*N*-1)] ^½^ Σ_*i*_|I_*i*_ (*hkl*) - < I(*hkl*) > |/Σ_*hkl*_Σ_*i*_ I_*i*_(*hkl*) [34][35]. ^‡‡‡^Figure of Merit is the probability of the phase angle to be correct. ^‡‡‡‡^Phasing Power is the sum of the anomalous contributions divided by the sum of the difference between the observed and calculated heavy atom derivative structure factor amplitudes. ^§^Typically, the number of unique reflections used in refinement is slightly less than the total number that were integrated and scaled. Reflections are excluded owing to negative intensities and rounding errors in the resolution limits and unit-cell parameters. ^¶^*R*_*cryst*_ = Σ_*hkl*_||*F*_obs_| - |*F*_calc_||/Σ_*hkl*_|*F*_obs_|, where *F*_calc_ and *F*_obs_ are the calculated and observed structure-factor amplitudes, respectively. *R*_*free*_ is the same as *R*_*cryst*_ but for 6991 reflections (5.0% of the total reflections chosen at random and omitted from refinement. ^††^This value represents the total *B* that includes TLS and residual *B* components. ^±^Percentage of residues in favored regions of Ramachandran plot (No. outliers in parenthesis). ^‡‡‡‡‡^Estimated overall coordinate error [36]. ^⟂⟂^One of the protein chains (Chain D) is modeled in two half-occupancy conformations. Click here for file

Additional file 2: Table S2 Structure and sequence based homology recognition analysis of the N-terminal of the BT_1012 protein (3KZS). This table shows the top hits for PF13204 (N-terminal domain of BT_1012, 3KZS) of DALI, FATCAT and FFAS searches against the PDB database. Click here for file

Additional file 3: Figure S1 A phylogenetic tree of the whole PF13204 family built by the average linkage method based on a MAFFT multiple sequence alignment. Bright green color identifies members with Asp and red block members with Glu at the donor site. Click here for file

Additional file 4: Table S4 Structure and sequence based homology recognition analysis of the full length BT_1012 protein (3KZS). This table shows the top hits of DALI, FATCAT and FFAS searches against the PDB database, only proteins with two or more domains are listed. Click here for file

Additional file 5: Table S3 Structure and sequence based homology recognition analysis of the C-terminal domain of the BT_1012 protein (3KZS). This table shows the top hits for PF12904 (C-terminal domain of BT_1012, 3KZS) of DALI, FATCAT and FFAS searches against the PDB database. Click here for file
