# Supplementary material for: Structural genomics analysis of uncharacterized protein families overrepresented in human gut bacteria identifies a novel glycoside hydrolase
Source: BMC Bioinformatics. 2014 Apr 17;15:112. doi: 10.1186/1471-2105-15-112 (PMC4032388; doi:10.1186/1471-2105-15-112)
Supplement: Additional file 1: Table S1 — Data collection and refinement statistics (PDB 3kzs). Values in parentheses are for the highest resolution shell. †R merge = Σ hkl Σ i |I i (hkl) - (I(hkl))|/Σ hkl Σ i (hkl). ‡R meas = Σ hkl [N/(N-1)]1/2Σ i |I i (hkl) - (I(hkl))|/Σ hkl Σ i I i (hkl)[33]. ‡‡R p.i.m (precision-indicating R merge ) = Σ hkl [(1/(N-1)] ½ Σ i |I i (hkl) - < I(hkl) > |/Σ hkl Σ i I i (hkl) [34][35]. ‡‡‡Figure of Merit is the probability of the phase angle to be correct. ‡‡‡‡Phasing Power is the sum of the anomalous contributions divided by the sum of the difference between the observed and calculated heavy atom derivative structure factor amplitudes. §Typically, the number of unique reflections used in refinement is slightly less than the total number that were integrated and scaled. Reflections are excluded owing to negative intensities and rounding errors in the resolution limits and unit-cell parameters. ¶R cryst = Σ hkl ||Fobs| - |Fcalc||/Σ hkl |Fobs|, where Fcalc and Fobs are the calculated and observed structure-factor amplitudes, respectively. R free is the same as R cryst but for 6991 reflections (5.0% of the total reflections chosen at random and omitted from refinement. ††This value represents the total B that includes TLS and residual B components. ±Percentage of residues in favored regions of Ramachandran plot (No. outliers in parenthesis). ‡‡‡‡‡Estimated overall coordinate error [36]. ⟂⟂One of the protein chains (Chain D) is modeled in two half-occupancy conformations. [file 1471-2105-15-112-S1.docx]

**Table S1. Data collection and refinement statistics (PDB 3kzs)**

| **Data collection** |  | | |
| --- | --- | --- | --- |
| Beamline | SSRL 11-1 | | |
| Space group/Unit cell | H32, a=b=261.34, c=183.87,c=59.7 Å, α=β=90°, γ=120° | | |
| Data | λ_1_ MADSe | λ_2_ MADSe | λ_3_ MADSe |
| Wavelength (Å) | 0.9116 | 0.9787 | 0.9781 |
| Resolution range (Å) | 85.12-2.10 | 85.13-2.10 | 85.13-2.10 |
| Data Collection Temperature (K) | 100 | 100 | 100 |
| Redundancy | 6.4 | 7.0 | 6.4 |
| No. of observations | 888,788 | 977,377 | 888,634 |
| No. of unique reflections | 139,171 | 139,348 | 139,400 |
| Completeness (%) | 100.0 (100.0) | 100.0(100.0) | 100.0 (199,9) |
| Mean I/σ (I) | 11.7 (2.2) | 11.2 (2.1) | 10.3(1.9) |
| R_merge_ on I (%)^†^ | 14.3(78.1) | 15.6 (88.1) | 16.5 (93.7) |
| R_meas_ on I (%)^‡^ | 15.6(84.9) | 16.9(95.2) | 17.9 (102.1) |
| R_pim_ on I (%)^‡‡^ | 6.1 (33.5) | 6.3 (35.8) | 7.0 (40.3) |
| Highest resolution shell | 2.15-2.10 | 2.15-2.10 | 2.15-2.10 |
| **Phasing Statistics** |  |  |  |
| Resolution Range (Å) | 50.0-2.10 |  |  |
| Figure of Merit^‡‡‡^ Acentric | 0.48 |  |  |
| Figure of Merit^‡‡‡^ Centric | 0.42 |  |  |
| Phasing Power^‡‡‡‡^ Isomorphous Acentric | 0.0 | 1.41 | 0.97 |
| Phasing Power^‡‡‡‡^ Isomorphous Centric | 0.0 | 1.28 | 0.90 |
| Phasing Power^‡‡‡‡^ Anomalous Acentric | 0.63 | 0.55 | 0.95 |
| Phasing Power^‡‡‡‡^ Anomalous Centric | 0.0 | 0.0 | 0.0 |
| **Model and refinement statistics** | | | |
| Data used in refinement | λ_1_MADSe |  | |
| No. of reflections (total) | 139,161 |  |  |
| No. of reflections (test) | 6,991 |  | |
| Cutoff criteria | F\|>0 |  | |
| R_cryst_ (%)^¶^ | 18.5 |  | |
| R_free_ (%)^¶^ | 22.2 |  | |
| **Stereochemical parameters** | | | |
| Restraints (RMSD observed) | | | |
| Bond Angles (Å) | 0.010 |  | |
| Bond angles (°) | 0.84 |  | |
| Ramachandran plot (%)^±^ | 94.1 (7) |  | |
| Rotamer outlier (%) | 0.78 |  | |
| Average isotropic B-value (total) (Å^2^) ^††^ | 27.9 |  | |
| Average isotropic B-value (protein) (Å^2^) ^††^ | 27.4 |  | |
| Average isotropic B-value (solvent) (Å^2^) ^††^ | 34.6 |  | |
| ESU based on R_free_ (Å)^‡‡‡‡‡^ | 0.186 |  | |
| No. protein atoms/residues / chains^⊥⊥^ | 14,947/2283/4 |  | |
| Non-protein entities | 16 SO_4_, 8 MRD, 2 MPD, 1208 H_2_O |  | |
