# Supplementary material for: Structural genomics analysis of uncharacterized protein families overrepresented in human gut bacteria identifies a novel glycoside hydrolase
Source: BMC Bioinformatics. 2014 Apr 17;15:112. doi: 10.1186/1471-2105-15-112 (PMC4032388; doi:10.1186/1471-2105-15-112)
Supplement: Additional file 2: Table S2 — Structure and sequence based homology recognition analysis of the N-terminal of the BT_1012 protein (3KZS). This table shows the top hits for PF13204 (N-terminal domain of BT_1012, 3KZS) of DALI, FATCAT and FFAS searches against the PDB database. [file 1471-2105-15-112-S2.pdf]

Table S2. Structure and sequence based homology recognition of N-terminal of 3KZS.

Chain	Z-score/ p value	Seq. id	GH family	GH superfamily	PDB NAME
			DALI		
A	19	17.0	GH5	7	ENDO-BETA-MANNANASE
A	18.1	18.0	GH5	7	ENDO-BETA-D-1,4-MANNANASE
A	18	17.0	GH5	7	ENDO-1,4-B-D-MANNANASE
A	17.6	18.0	GH5	7	GH5 ENDO-BETA-1,4-MANNANASE
A	16.8	17.0	GH5	7	MANNOSYL-OLIGOSACCHARIDE GLUCOSID
A	16.7	18.0	GH5	7	MANNAN ENDO-1,4-BETA-MANNOSIDASE.
A	16.7	16.0	GH5	10	MANNAN ENDO-1,4-BETA-MANNOSIDASE
A	16.5	14.0	GH5	2	ENDOGLUCANASE 5A
A	16.5	15.0	GH5	10	BETA-1,4-MANNANASE
A	16.4	12.0	GH5	2	ENDOGLUCANASE
			FATCAT		
A	5.71E-11	13.2	GH5	7	ENDO-BETA-MANNANASE

A	2.26E-09	12.5	GH5	7	MANNOSYL-OLIGOSACCHARIDE GLUCOSID
A	2.31E-09	11.7	GH5	2	NATIVE ENDOGLUCANASE
A	4.41E-09	12.0	GH5	8	PROTEIN (BETA-MANNANASE)
A	5.95E-09	9.7	GH2		BETA-GALACTOSIDASE
A	6.98E-09	10.5	GH2		BETA-GLUCURONIDASE
A	1.35E-08	9.2	GH5	2	CELLULASE
A	1.71E-08	11.7	GH5	10	MANNAN ENDO-1,4-BETA-MANNOSIDASE
A	2.54E-08	8.4	GH5	5	THE MAJOR ENDOGLUCANASE
A	1.15E-07	8.8	GH53		BETA-1,4-GALACTANASE
**FFAS/PDB**					
A	-54.5	15.0	GH5	36	GH5 ENDO-BETA-1,4-MANNANASE
A	-53.3	15.0	GH5	7	ENDO-BETA-D-1,4-MANNANASE
A	-52.9	15.0	GH5	7	ENDO-1,4-B-D-MANNANASE
A	-50.3	15.0	GH5	7	MANNOSYL-OLIGOSACCHARIDE GLUCOSIDASE

A	-48.9	16.0	GH5	7	ENDO-1,4-B-D-MANNANASE
A	-48.6	16.0	GH5	7	MANNAN ENDO-1,4-BETA-MANNOSIDASE
A	-48.3	13.0	GH5	7	MANNAN ENDO-1,4-BETA-MANNOSIDASE
A	-47.9	11.0	GH5	7	BETA-1,4-MANNANASE
A	-44.3	13.0	GH5	0	ENDOCELLULASE E1
A	-41.2	11.0	GH5	10	ENDOGLYCOCERAMIDASE II
