# Supplementary figures and images for: Structural genomics analysis of uncharacterized protein families overrepresented in human gut bacteria identifies a novel glycoside hydrolase
Source: BMC Bioinformatics. 2014 Apr 17;15:112. doi: 10.1186/1471-2105-15-112 (PMC4032388; doi:10.1186/1471-2105-15-112)

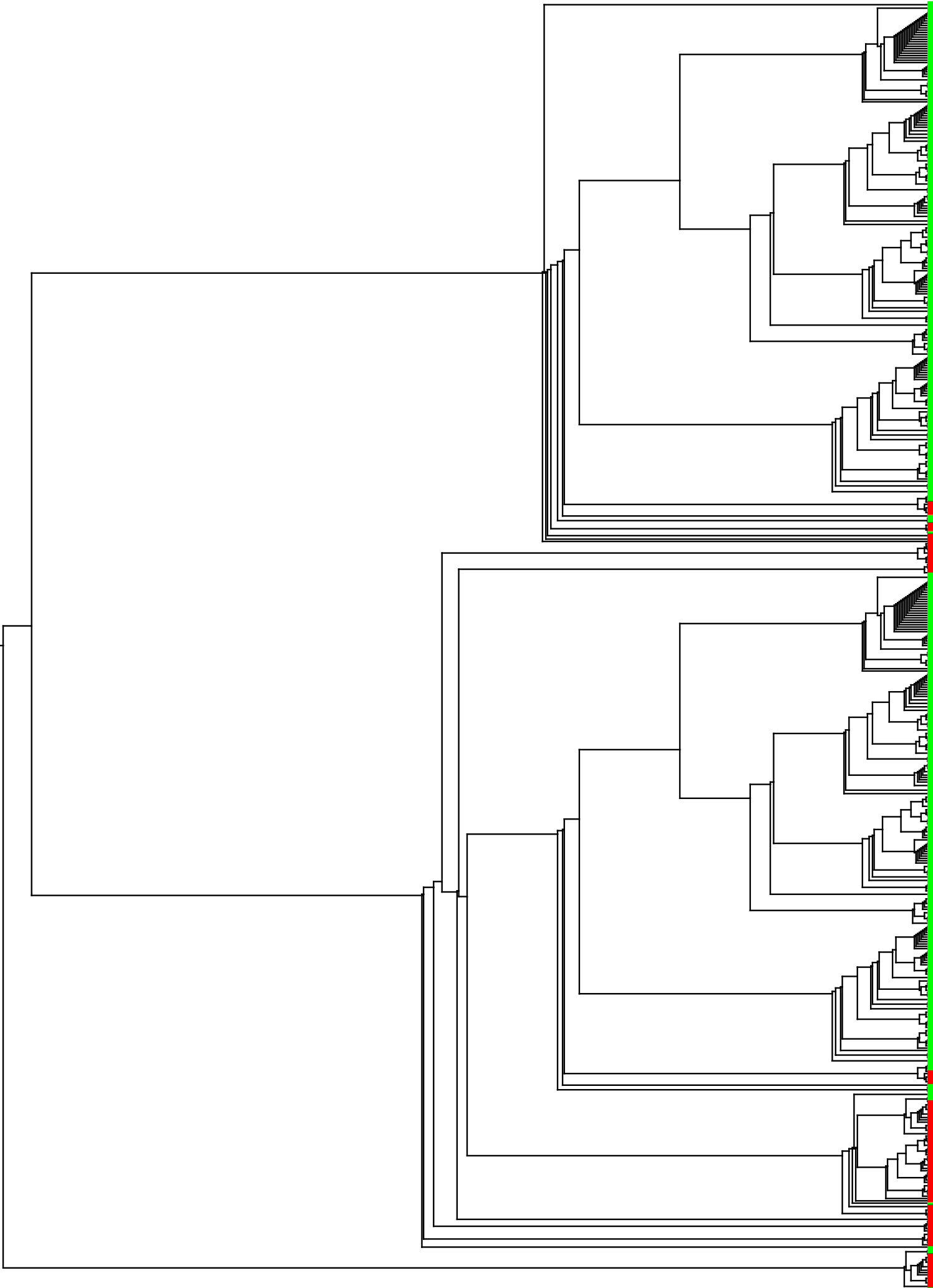

Supplement: Additional file 3: Figure S1 — A phylogenetic tree of the whole PF13204 family built by the average linkage method based on a MAFFT multiple sequence alignment. Bright green color identifies members with Asp and red block members with Glu at the donor site. [file 1471-2105-15-112-S3.pdf]
