# Supplementary material for: Structural genomics analysis of uncharacterized protein families overrepresented in human gut bacteria identifies a novel glycoside hydrolase
Source: BMC Bioinformatics. 2014 Apr 17;15:112. doi: 10.1186/1471-2105-15-112 (PMC4032388; doi:10.1186/1471-2105-15-112)
Supplement: Additional file 4: Table S4 — Structure and sequence based homology recognition analysis of the full length BT_1012 protein (3KZS). This table shows the top hits of DALI, FATCAT and FFAS searches against the PDB database, only proteins with two or more domains are listed. [file 1471-2105-15-112-S4.pdf]

Table S4. Structure and sequence based homology recognition of 3KZS.

PDBID	Chain	Z-score/ p value	Seq. id	GH family	PDB NAME	Corresponding domain	Function {PMID}
				DALI			
3CMG	A	20	12.0	GH2	PUTATIVE BETA-GALACTOSIDASE	PF02837/PF00703/PF02836	N/A
3OG2	A	20.1	10.0	GH35	BETA-GALACTOSIDASE	PF13364/PF01301/PF10435/PF13364/PF13363	The only catalytic domain surrounded by horseshoe consisting of five anti-parallel β-sandwich structures {21130883}
1TG7	A	20.1	11.0	GH35	BETA-GALACTOSIDASE	PF13364/PF13364/PF10435/PF13363/PF01301	The only catalytic domain surrounded by four anti-parallel β-sandwich structure{15491613}
3U7V	A	20.4	12.0	GH35	BETA-GALACTOSIDASE	PF13364/PF13364	N/A
3OB8	A	18	13.0	GH2	BETA-GALACTOSIDASE	PF00703/PF02836/PF02929/PF02837/	The only catalytic domain surrounded by four β-sandwich and j-roll domains. The two of them are building up the pocket entrance.{ 22193516}
3BGA	A	18	12.0	GH2	BETA-GALACTOSIDASE	PF02929/PF02837/PF00703/PF02836	N/A
41976	A	18.3	12.0	GH2	BETA-GALACTOSIDASE	N/A	N/A
3TTS	A	21.5	14.0	GH42	BETA-GALACTOSIDASE	PF02449/PF08532/PF08533	The catalytic domain with two auxiliary domains {22385475 }
1WKY	A	19.8	13.0	GH5_8 / CBM59	ENDO-BETA-1,4-MANNANASE	PF00150	Two domain structure where C-terminal doamin is non catalytic CBM59 {15272186}

1H3G	A	11.4	8.0	N/A	CYCLOMALTODEXTRINASE	PF09087/PF10438/PF00128	The catalytic domain surrounded by β-sandwich and the b-barrel domains {12752453}
**FATCAT**							
1KWG	A	2.2E-08	9.9	GH42	BETA-GALACTOSIDASE	PF02449/PF08532/PF08533/PF01373	Three domain structure (and one subdomain) , where the catalytic domain followed by a starch-binding domain { 12215416}
1VFF	A	2.3E-07	5.2	GH1	BETA-GLYCOSIDASE	PF02449/PF08532	N/A
1E4M	A	0.0035	5.1	GH1	BETA-AMYLASE	PF02449/PF08532/PF08533/PF01373	N/A
1VEM	A	0.0035	5.1	GH14 / CBM20	BETA-AMYLASE	PF00686/PF02449/PF01373/PF02806	Two domain structure {15449941}
1WZA	A	0.00586	4.0	GH13	ALPHA-AMYLASE	N/A	Three domain structure, one catalytic and two non-catalytic domains {16647060}
1LWH	A	0.0159	5.2	GH13	4-ALPHA-GLUCANOTRANSFERASE	PF09178/PF00128	Three domain structure, one catalytic and two non-catalitis domains {12139940}
1XRS	A	0.0306	6.3	N/A	LYSINE 5,6-AMINOMUTASE	PF02310/PF09043	Two domain structure: catalytic domain and substrate binding domain {15514022}
3BOF	A	1.6E-07	6.7	N/A	COBALAMIN-DEPENDENT METHIONINE SYNTHASE	PF00809/PF02574	Two doamins: Hcy-binding domain and catalytic domain {18296644}

1JI1	A	0.0337	5.3	GH13 / CBM34	ALPHA-AMYLASE 1	PF00128/PF02903	Two domain structure: C-terminal domain is non catalytic { 12051850 }
1PS9	A	0.00041	6.4	N/A	2,4-DIENOYL-COA REDUCTASE	PF00724/PF07992/PF03486/PF13738/PF13450	N/A
**FFAS/PDB**							
2Y8K	A	-36.7	10	GH5_34 / CBM6	ARABINOXYLAN-SPECIFIC XYLANASE	PF00150/PF03422	Two domains: catalytic and noncatalytic carbohydrate-binding module {21378160}
2OSW	A	-35.3	9	GH5_28	ENDO-GLYCOCERAMIDASE II	PF00150/PF02449	Two domain structure, C-terminal is not involved in substrate binding {17329247 }
4EKJ	A	-22.7	9	GH39	BETA-XYLOSIDASE	PF00150/PF01229	C-terminal domain is the auxiliary domain that modifies the catalytic interface {22993088}
1W91	A	-21.6	8	GH39	BETA-XYLOSIDASE	PF01229	N/A
1PX8	A	-20.9	9	GH39	BETA-XYLOSIDASE	PF01229/PF11790	C-terminal domain is the auxiliary domain that modifies the catalytic interface { 14659747}
4JXO	A	-18.4	8	GH39	ALPHA-L-IDURONIDASE	N/A	N/A
1KWG	A	-15.7	11	GH42	BETA-GALACTOSIDASE	PF02449/PF08532/PF08533/PF01373	Three domain structure (and one subdomain), the catalytic domain is followed by a starch-binding domain {12215416}

3GM8	A	-15.3	10	GH2	GLYCOSIDE HYDROLASE FAMILY	PF02836/PF02837/PF00703	N/A
2YIH	A	-14.3	9	GH44	XYLOGLUCANASE	PF12891	Three domain structure, function of the non-catalytic domain is unclear {21795708}
3U7V	A	-14	10	GH35	BETA-GALACTOSIDASE	PF13364/PF13364	N/A
