# Supplementary material for: Structural genomics analysis of uncharacterized protein families overrepresented in human gut bacteria identifies a novel glycoside hydrolase
Source: BMC Bioinformatics. 2014 Apr 17;15:112. doi: 10.1186/1471-2105-15-112 (PMC4032388; doi:10.1186/1471-2105-15-112)
Supplement: Additional file 5: Table S3 — Structure and sequence based homology recognition analysis of the C-terminal domain of the BT_1012 protein (3KZS). This table shows the top hits for PF12904 (C-terminal domain of BT_1012, 3KZS) of DALI, FATCAT and FFAS searches against the PDB database. [file 1471-2105-15-112-S5.pdf]

Table S3. Structure and sequence based homology recognition of C-terminal of 3KZS.

PDBID	Chain	Z-score/	Seq. id	GH family	PDB NAME	Corresponding domain	Function of corresponding domain {PMID}
					DALI		
3A24	A	7.3	17.0	GH97	ALPHA-GALACTOSIDASE	C-terminal domain of three domain structure	Non catalytic { 19646996 }
3LXB	B	7.2	4.0	GH27	ALPHA-GALACTOSIDASE A	C-terminal domain of two domain structure	Non catalytic { 20444686}
1PX8	A	6.3	3.0	GH39	BETA-XYLOSIDASE	C-terminal domain of three domain structure	Auxiliary { 14659747}
3ZR5	A	6.3	3.0	GH59	GALACTOCEREBROSIDASE	C-terminal domain of two domain structure	Non catalytic but form the cleavage site { 21876145}
2D73	A	6.3	13.0	GH97	ALPHA-GLUCOSIDASE SUSB	C-terminal domain of two domain structure	Stabilizing the catalytic domain { 18981178}
3VMN	A	6.1	7.0	GH66	DEXTRANASE	C-terminal domain of two domain structure	Unclear { 22337884}
2BFG	A	6.1	8.0	GH39	BETA-XYLOSIDASE	C-terminal domain of two domain structure	Non catalytic { 16212978}
4JXO	B	6	7.0	GH39	ALPHA-L-IDURONIDASE	C-terminal part of a one domain protein	N/A
3IK2	A	5.9	2.0	GH44	ENDOGLUCANASE A	C-terminal domain of two domain structure	Non catalytic{ 19915043}
2YFN	A	5.9	6.0	N/A	ALPHA-GALACTOSIDASE-SUCROSE KINASE AGASK	C-terminal domain of three domain structure	Catalyzes the phosphorylation of the sucrose{ 21931163}

FATCAT							
1R46	A	0.0003	4.6	GH27	ALPHA-GALACTOSIDASE A	C-terminal domain of two domain structure	Non catalytic { 15003450 }
1NOF	A	0.00079	8.3	GH30	XYLANASE	C-terminal domain of two domain structure	Form the cleavage site { 12859186 }
1JI1	A	0.00117	3.1	GH13 / CBM34	ALPHA-AMYLASE I	C-terminal domain of three domain structure	CBM { 12051850 }
1SZN	A	0.00156	7.1	GH27	ALPHA-GALACTOSIDASE	C-terminal domain of two domain structure	Unclear { 15136043}
1DEC	A	0.00174	7.6	N/A	DECORSIN	decorsin	Collagen binding { 8009227 }
1UHV	A	0.00206	3.6	GH39	BETA-XYLOSIDASE	C-terminal domain of three domain structure	Non catalytic { 11527972}
1F1S	A	0.00222	5.0	PL8	HYALURONATE LYASE	C-terminal domain of three domain structure	Non catalytic { 11527972}
1BF2	A	0.00243	8.7	GH13 / CBM48	ISOAMYLASE	C-terminal domain of three domain structure	CBM { 9719642 }
1HX0	A	0.00268	8.6	GH13	ALPHA AMYLASE (PPA)	C-terminal domain of three domain structure	Non catalytic { 11412124 }

1N7O	A	0.00296	4.8	PL8	HYALURONATE LYASE	C-terminal domain of two domain structure	Non catalytic domain , controls the access to the cleft { 12446724}
**FFAS/PDB**							
4FQ1	L	-10.4	11.0	N/A	FAB HEAVY CHAIN	light-chain	Might be involved in contact regions for epitopes { 23115339 }
4JY5	L	-10.1	14.0	N/A	PGT122 LIGHT CHAIN	light-chain	Might be involved in glycan accommodation {23658524}
4FQ2	L	-9.51	11.0	N/A	FAB HEAVY CHAIN	light-chain	Might be involved in contact regions for epitopes { 23115339}
